# Correlation between epidermal growth factor receptor and tumor stem cell markers CD44/CD24 and their relationship with prognosis in breast invasive ductal carcinoma

**DOI:** 10.1007/s12032-014-0275-2

**Published:** 2014-11-28

**Authors:** Zhousan Zheng, Nan Shao, Huiwen Weng, Wen Li, Jiaxing Zhang, Luanjing Zhang, Lily Yang, Sheng Ye

**Affiliations:** 1 Department of Oncology, the First Affiliated Hospital of Sun Yat-sen University, 58th Zhongshan Second Road, Guangzhou, 510080 Guangdong province China; 2 Department of Breast Surgery, the First Affiliated Hospital of Sun Yat-sen University, Guangzhou, People’s Republic of China; 3 Department of Surgical Laboratory, the First Affiliated Hospital of Sun Yat-sen University, Guangzhou, People’s Republic of China; 4 Departments of Surgery and Radiology, Winship Cancer Institute, Emory University, School of Medicine, Atlanta, GA USA

**Keywords:** Breast infiltrating ductal carcinoma, EGFR, CD44, CD24, Prognosis

## Abstract

We studied the correlation between epidermal growth factor receptor (EGFR) and the tumor stem cell markers CD44/CD24 in breast invasive ductal carcinoma (BIDC) and their relationship with prognosis. We analyzed the clinical data of 139 BIDC cases retrospectively, detecting EGFR, CD44, and CD24 expressions in tumor tissue using immunohistochemistry. The proportion of EGFR-, CD44-, and CD24-positive cases was 59.0, 62.3, and 30.9 %, respectively. The proportion of CD44-positive [76.9 % (*p* < 0.05)] and EGFR-positive [67.2 % (*p* = 0.108)] cases in the triple-negative breast cancer (TNBC) group was higher than that of the non-TNBC group. In the non-TNBC group, 36.5 % was CD24-positive, higher than that in the TNBC group but not statistically significant. The proportion of CD44-positive cases was significantly higher in the EGFR-positive group than in the EGFR-negative group (*p* = 0.017). EGFR-positive cases were significantly correlated with premenopausal status (*p* = 0.036), distant metastasis (*p* = 0.018), and estrogen receptor-negative status (*p* = 0.020). CD44-positive status was significantly correlated with human epidermal growth receptor 2 (HER2)-negative (*p* = 0.023), estrogen receptor-negative (*p* = 0.021), and progesterone receptor-negative status (*p* = 0.004). CD24-positive status was significantly correlated with HER2-positive status (*p* = 0.001). Kaplan–Meier survival analysis showed that TNBC patients had shorter survival. EGFR-positive and CD44-positive status were both correlated with shorter survival in the lymph node- and HR-negative groups, while CD24 positive was significantly correlated with poor survival in lymph node-negative and HR-positive patients. EGFR and CD44 expressions have a significantly positive correlation (*p* = 0.017) in BIDC. Patients both EGFR and CD44 positive had the worst outcome.

## Introduction

Breast cancer is a heterogeneous disease with different molecular profiles, clinical behaviors, and responses to therapy. Modern genomic and immunohistochemical techniques have enabled the classification of breast cancers into distinct subsets, including hormone receptor positive (luminal A and luminal B), human epidermal growth receptor 2 (HER2)-positive, and basal-like type [1]. Triple-negative breast cancer (TNBC) accounts for 90 % of the basal-like type and is characterized by its biological aggressiveness, worse prognosis, and lack of therapeutic target in contrast with hormone receptor-positive and HER2-positive breast cancer [2, 3]. Immunohistochemically, TNBC is typically negative for estrogen receptor (ER), progesterone receptor (PR), and HER2, but positive for basal cytokeratins (CK5/6/14/17), epidermal growth factor receptor (EGFR), and/or c-kit [4, 5].

EGFR is a cell surface receptor, and its expression has been implicated in multiple biological processes, including proliferation, apoptosis, invasion, and angiogenesis, which are the hallmarks of cancer [6]. Using gene and protein expressions, previous studies have reported high EGFR expression in most TNBC [7, 8]. High expression of EGFR is reported to be associated with poor clinical outcome in breast cancer, while its prognostic value remains debated [9, 10].

The stem cell-like phenotype of tumor-initiating cells and their limited number within the bulk of a tumor may account for their ability to escape conventional therapies, leading to disease relapse although the primary lesion has been removed [11]. In breast cancer, Al-Hajj et al. [12] were the first to isolate a highly tumorigenic subpopulation of tumor cells with the CD44+/CD24− phenotype. They demonstrated that CD44+/CD24− tumor cells resembled normal stem/progenitor cells with respect to their ability to self-renew, proliferate, and differentiate [11]. Although only one-third of human breast cancers have the CD44+/CD24− phenotype, this tumor cell population appears most commonly in TNBC [13].

Research on the correlation between EGFR expression and CD44/CD24 and their prognostic value in breast invasive ductal carcinoma (BIDC) is limited. In this study, we aimed to evaluate the correlation between EGFR expression and CD44/CD24 and determine their relationship with BIDC clinicopathological parameters and their prognostic value in BIDC.

## Materials and methods

### Patients and samples

We enrolled 765 patients who had undergone surgery for primary breast cancer at the First Affiliated Hospital of Sun Yat-Sen University from January 2000 to December 2005. Of these, 65 (8.5 %) had TNBC. We selected another 74 luminal or HER2-positive patients (non-TNBC) randomly as the control. All patients were diagnosed as BIDC by immunohistochemistry (IHC). All patients underwent radical mastectomy, modified radical mastectomy, breast-conserving surgery, or mastectomy. None had received chemotherapy or radiotherapy before surgery. Adjuvant systematic therapy (chemotherapy, radiotherapy, endocrine therapy) was administered as clinically indicated in accordance with standard practices during this interval. Clinicopathological information was obtained by reviewing medical records and pathology reports. We obtained the following variables: age; tumor size; Bloom-Richardson histological grade; lymph node status; and ER, PR, and HER2 status. The Ethics Committee of the First Affiliated Hospital of Sun Yat-Sen University reviewed and approved this study, which was performed in accordance with the ethical standards described in the Declaration of Helsinki. Overall follow-up was calculated from the date of surgery to the date of the last follow-up (April 2013) or breast cancer-related death. The median follow-up period was 97 months (2–156 months).

### Immunohistochemistry

Paraffin-embedded primary tumor tissue was retrieved from the First Affiliated Hospital of Sun Yat-Sen University Department of Pathology, from which we obtained 4-μm-thick sections. We used the DAKO EnVision system (DAKO EnVision labeled polymer, peroxidase; Dako, Glostrup, Denmark) to detect CD44 (1:200; ZSGB-bio, Beijing, China), CD24 (1:200; ZSGB-bio), and EGFR (1:200; ZSGB-bio). The appropriate positive controls were used. Phosphate-buffered saline was substituted for the primary antibody as the negative control. The color was developed through incubation with 3, 3′-diaminobenzidine.

Two pathologists scored the proportion of positively stained tumor cells and staining intensity independently, and a consensus score was given for each case. CD44 and CD24 staining was detected mainly in the cell membrane and occasionally in the cytoplasm. According to Wu et al. [14], the intensity of positive staining was scored as follows: (+++) for high-intensity staining, (++) for moderate staining, (+) for low intensity, and (−) for no staining. The percentage of stained cells was categorized as 0 (negative), 1 (<10 % positive cells), 2 (11–50 % positive cells), 3 (51–80 % positive cells), and 4 (80–100 % positive cells). The final quantification of IHC results for both variables (staining intensity and percentage of positively stained cells) was considered (score = staining intensity × positive staining).

EGFR staining was detected mainly in the cell membrane and cytoplasm. The proportion of positively stained cells was scored as follows: 0 (no positive cells), 1 (<25 % positive cells), 2 (26–50 % positive cells), 3 (50–75 % positive cells), and 4 (>75 % positive cells). Staining intensity was graded as follows: 0 (no staining), 1 (weak staining = light yellow), 2 (moderate staining = yellow brown), and 3 (strong staining = brown). The staining index was calculated as the product of the staining intensity score and proportion of positive cells. A staining index score of ≤6 indicated negative expression; a staining index score of >6 indicated positive expression.

### Statistical analyses

Disease-free survival (DFS) was defined as the duration from the date of primary surgery to the first local recurrence or distant metastasis. Overall survival (OS) was the duration from the date of primary surgery to the time of breast cancer-related death or the last follow-up.

Statistical analysis was performed using SPSS 17.0 statistical software (SPSS Inc., Chicago, IL, USA). The Chi-square test was used to analyze the relationship between EGFR, CD44/CD24 expressions, and clinicopathological variables. The association with survival was analyzed using the Kaplan–Meier plot and log-rank test. Survival data were evaluated using univariate and multivariate Cox regression analyses to adjust for other prognostic indicators. A *p* value of 0.05 was considered statistically significant.

## Results

### Tumor clinicopathological features

In total, we included 139 BIDC patients (TNBC, 65 cases; non-TNBC, 74 cases) in this study and analyzed their EGFR, CD44, and CD24 expressions. Table 1 summarizes the patient and tumor characteristics. All patients were all female; the median age was 50 years (range, 28–82 years).

**Table 1:** Patient characteristics

Parameters	Patients, *n* (%)	
	TNBC (*n* = 65) (%)	Non-TNBC (*n* = 74) (%)
Age group		
<35years	7 (10.8)	9 (12.2)
≥35years	58 (89.2)	65 (87.8)
Tumor size		
<3cm	30 (46.2)	44 (59.5)
≥3cm	35 (53.8)	30 (40.5)
Lymph node		
Positive	33 (50.8)	28 (37.8)
Negative	32 (49.2)	46 (62.2)
Menopausal		
Premenopausal	31 (47.7)	35 (47.3)
Postmenopausal	34 (52.3)	39 (52.7)
Pathological stage		
III	37 (56.9)	20(27.0)
I–II	28(43.1)	54(73.0)
Surgery		
Radical mastectomy	35 (53.8)	8 (10.8)
Modified radical mastectomy	22 (33.8)	62 (83.8)
Mastectomy	3 (4.6)	2(2.7)
Breast-conserving	5 (5.8)	2 (2.7)

### EGFR, CD44, and CD24 expressions

**Fig. 1:**
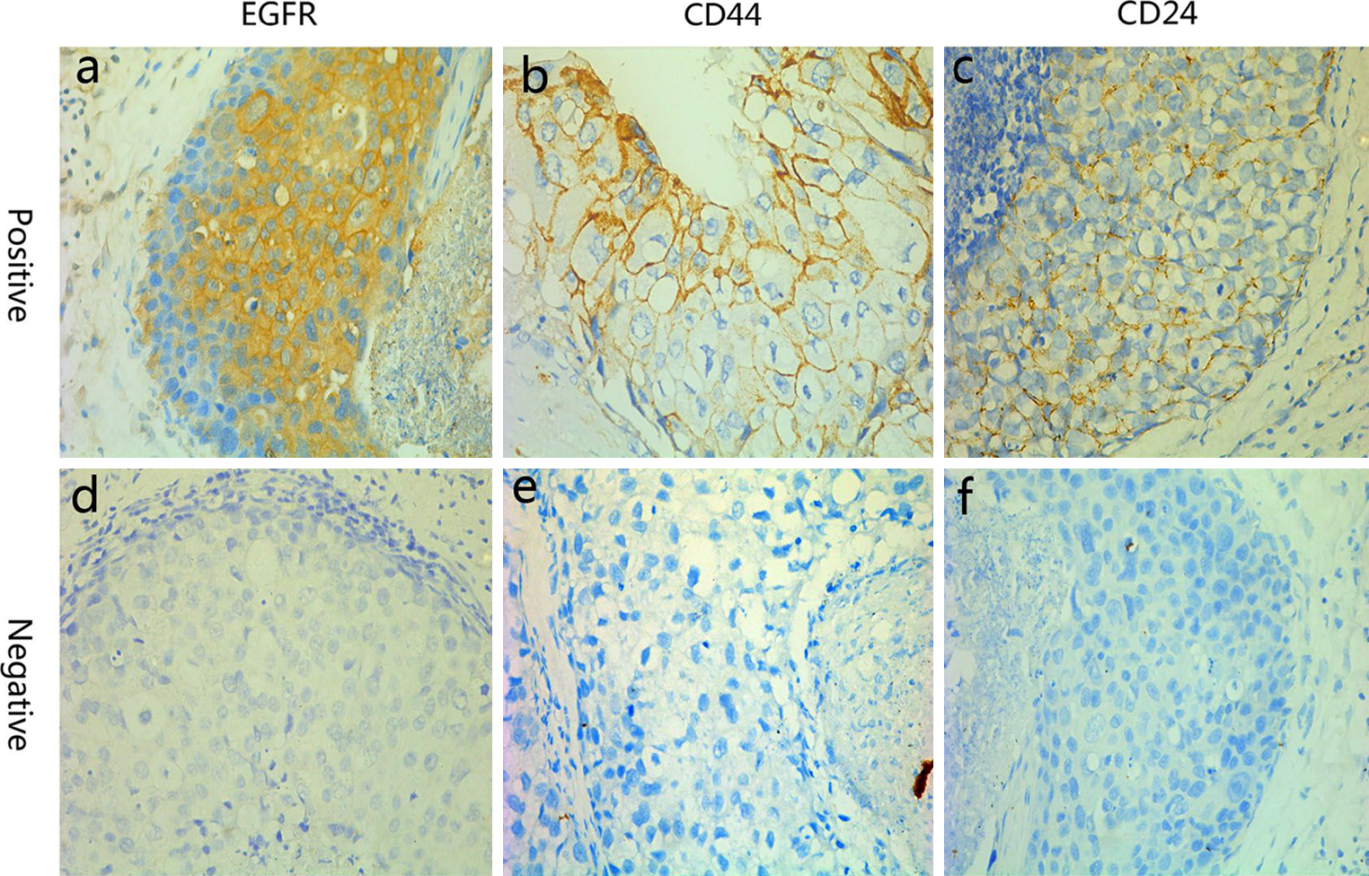
**a** Positive staining of EGFR in the cell membrane and cytoplasm. **b**, **c** CD44 and CD24 expression in breast cancer cells in the membrane and occasionally in the cytoplasm(x400)

Positive IHC staining of EGFR was detected in the cell membrane and cytoplasm in 82 cases (59.0 %; Fig. 1a). Positive CD44 and CD24 expressions were detected in the cell membrane and occasionally in the cytoplasm (Fig. 1b, c). There were positive CD44 and CD24 expressions in 87/139 (62.6 %) and 43/139 cases (30.9 %), respectively. Table 2 details the EGFR, CD44, and CD24 expressions in the TNBC and non-TNBC cases. More EGFR-positive patients were also CD44-positive and CD44+/CD24− subtypes compared to EGFR-negative patients (*p* = 0.017 and *p* = 0.037, respectively; Table 3); CD24 expression between the two groups was not statistically significant (*p* = 0.89). EGFR expression was associated with menopausal (*p* = 0.036) and ER-positive status (*p* = 0.020), CD44 expression was associated with HER2-positive (*p* = 0.023), ER-positive (*p* = 0.021) and PR-positive status (*p* = 0.004), and CD24 expression was associated with HER2-positive status (*p* = 0.001). Patients who were EGFR and CD44 positive were more likely to develop distant metastases (*p* = 0.018 and *p* = 0.064, respectively; Table 4).

**Table 2:** Marker expression

Parameters	All patients *n*=139	TNBC *n*=65	Non-TNBC *n*=74	X^2^	*p* value
EGFR					
Positive	82 (59.0)	43 (66.2)	39 (52.7)	2.588	0.108
Negative	57 (41.0)	22 (33.8)	35 (47.3)		
CD44					
Positive	87 (62.6)	50(76.9)	37(50)	10.712	0.001*
Negative	52 (37.4)	15(23.1)	37(50)		
CD24					
Positive	43 (30.9)	16(24.6)	27(36.5)	2.282	0.131
Negative	96 (69.1)	49(75.4)	47(63.5)		
CD44+/CD24−					
Yes	61(43.9)	36(55.4)	25(33.7)	6.557	0.010*
No	78(56.1)	29(44.6)	49(66.3)		

* *p* < 0.05

**Table 3:** Correlation between EGFR and CD44/CD24 expression, *n* (%)

	EGFR-positive *n*=82	EGFR-negative *n*=57	*p* value
CD44			
Positive	58 (70.7)	28 (49.1)	0.017*
Negative	24 (29.3)	29 (50.9)	
CD24			
Positive	25 (30.5)	18 (31.6)	0.891
Negative	57 (69.5)	39 (68.4)	
CD44+/CD24−			
Yes	42 (51.2)	19 (33.3)	0.037*
No	40 (48.8)	38 (66.7)	

* *p* < 0.05

**Table 4:** Correlation between marker expression and clinicopathological parameters

Parameters	EGFR		*p* value	CD44		*p* value	CD24		*p* value
	−	+		−	+		−	+	
Tumor grade			0.116			0.392			0.295
G1	4	16		7	13		16	4	
G2	27	35		27	35		39	23	
G3	26	31		18	39		41	16	
Age			0.166			0.994			0.585
<35 years	4	12		6	10		12	4	
≥35 years	53	70		46	77		84	39	
Menopausal status			0.036*			0.345			0.374
Premenopausal	21	45		22	44		48	18	
Postmenopausal	36	37		30	43		48	25	
Distant metastasis			0.018*			0.064			0.249
Yes	44	46		37	53		64	26	
No	11	30		10	31		25	16	
Censored	2	6		5	3		7	1	
Lymph node metastasis			0.724			0.261			0.489
Negative	33	45		26	52		52	26	
Positive	24	37		26	35		44	17	
Tumor size			0.567			0.554			0.487
<3cm	32	42		26	48		53	21	
≥3cm	25	40		26	39		43	22	
HER2 status			0.768			0.023*			0.001*
Negative	45	63		35	73		82	27	
Positive	12	19		17	14		14	16	
ER status									
Negative	30	59	0.020*	27	62	0.021*	63	26	0.558
Positive	27	23		25	25		33	17	
PR status									
Negative	28	50	0.166	21	57	0.004*	55	23	0.676
Positive	29	32		31	30		41	20	

* *p* < 0.05

### Survival analysis

Univariate analysis showed that TNBC patients had significantly worse DFS (*p* < 0.001) and OS (*p* = 0.001) than non-TNBC patients. The same was observed in patients who were EGFR positive (DFS: *p* = 0.002, OS: *p* = 0.003) and CD44 positive (DFS: *p* = 0.007, OS: *p* = 0.034). However, there was no significant difference between DFS and OS in CD24-positive and CD24-negative patients. Multivariate analysis for all patients indicated that TNBC and positive EGFR staining were significant prognostic factors for DFS and OS. (Table 5).

**Table 5:** Multivariate analysis

Parameters	DFS			OS		
	Hazard ratio	*p* value	95 % CI	Hazard ratio	*p* value	95 % CI
Tumor size (≥3cm)	1.566	0.153	0.846–2.897	1.830	0.071	0.950–3.526
Lymph node metastases	1.703	0.090	0.920–3.152	1.548	0.177	0.820–2.921
EGFR positive	2.255	0.023*	1.116–4.555	2.449	0.016*	1.180–5.080
CD44 positive	1.555	0.225	0.762–3.173	1.929	0.109	0.864–4.308
TNBC	2.088	0.023*	1.105–3.946	2.198	0.021*	1.124–4.295

* *p* < 0.05

**Fig. 2:**
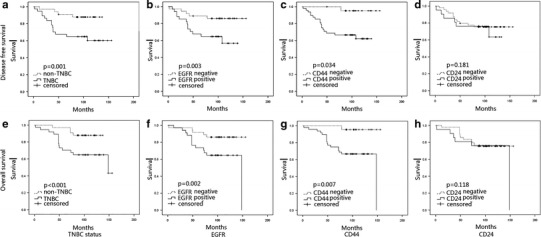
**a**, **e** TNBC-, **b**, **f** EGFR-, and **c**, **g** CD44-positive patients had significantly worse DFS and OS. **d**, **h** There was no significant difference in DFS and OS between CD24-positive and CD24-negative patients

**Fig. 3:**
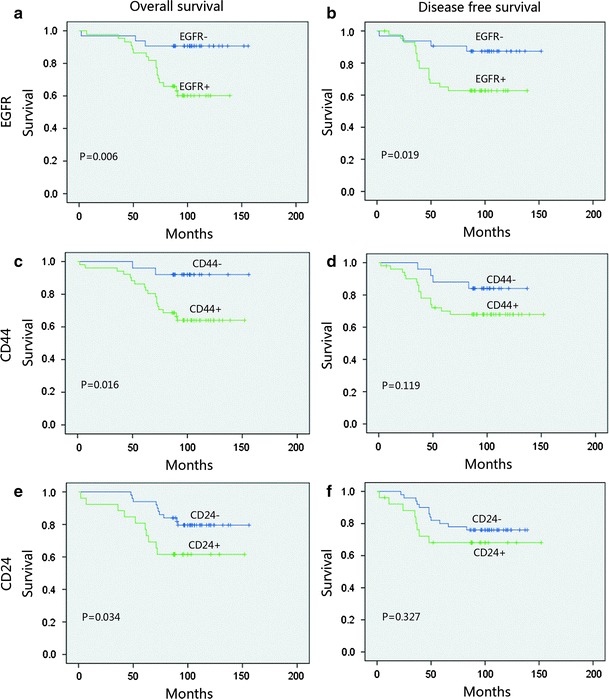
In node-negative patients, EGFR-positive patients DFS and OS were significantly lower than EGFR-negative patients (**a**, **b**) ; CD44- and CD24-positive both showed a significant negative correlation with OS (**c**, **e**)

**Fig. 4:**
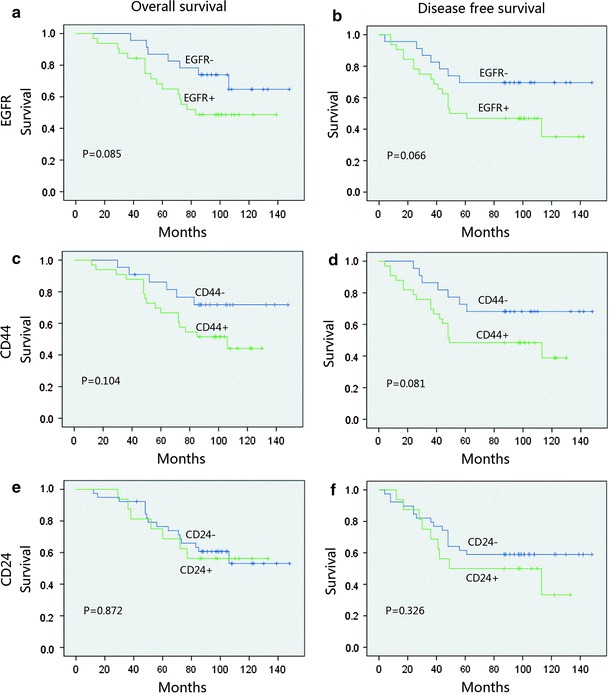
In node-positive patients, EGFR (**a**, **b**), CD44 (**c**, **d**) and CD24 (**e**, **f**) did not show a significant relationship with the OS and DFS

**Fig. 5:**
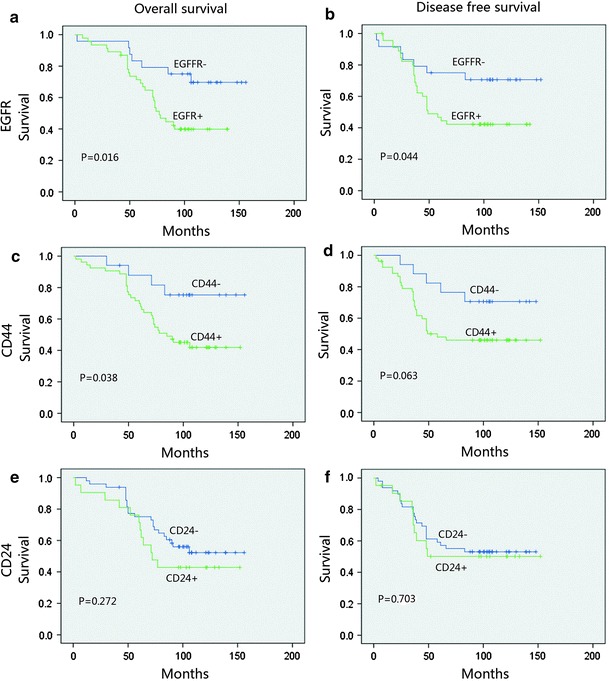
In HR (hormone receptor) negative patients, EGFR-(**a**, **b**) and CD44-(**c**, **d**) positive patients both experienced shorter OS and DFS

**Fig. 6:**
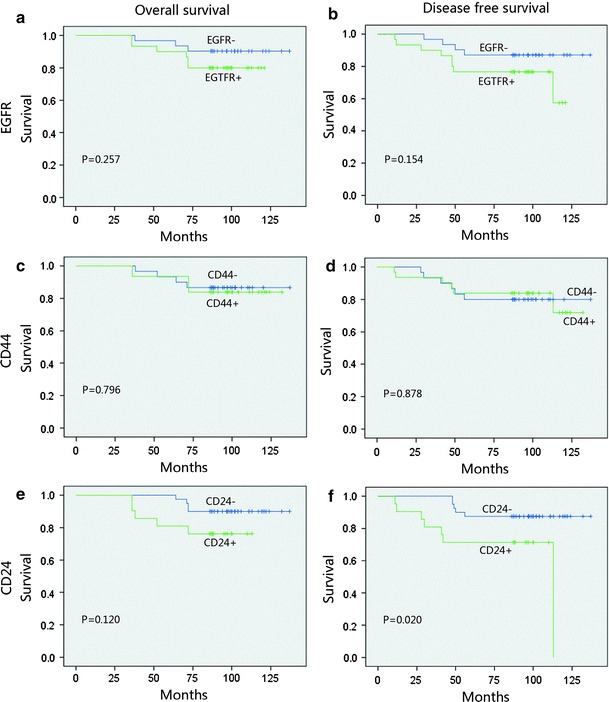
In HR-positive subtype, CD24-positive patients had significantly worse DFS (**f**)

**Fig. 7:**
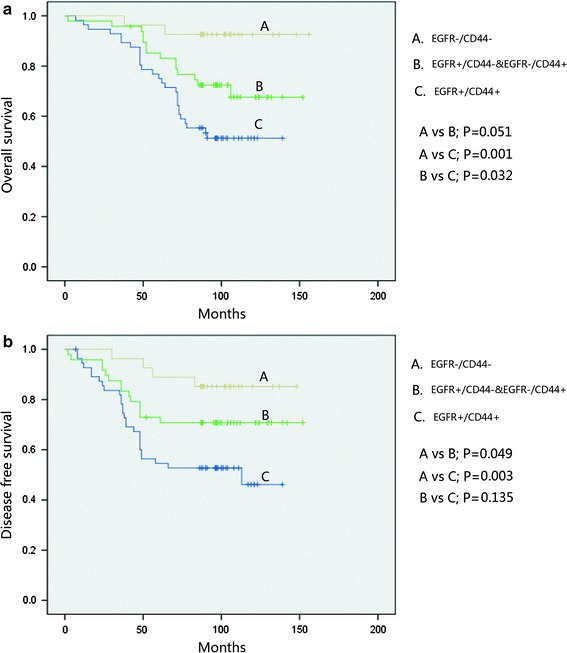
In EGFR/CD44 stratified analysis, patients with EGFR+/CD44+ subtype had the shortest OS and DFS, patients with EGFR/CD44 single positive subtype followed, patients with EGFR-/CD44- subtype had the longest OS and DFS (**a**, **b**)

Figure 2 depicts the survival curves. In stratified analysis, in node-negative patients and EGFR-positive patients, DFS and OS were significantly shorter than those in EGFR-negative patients (*p* = 0.019 and *p* = 0.006); CD44 and CD24 positive both showed an inferior OS (*p* < 0.05, Fig. 3). In node-positive patients, EGFR, CD44, and CD24 expressions did not show a significant relationship with the DFS and OS, (Fig. 4). In HR (hormone receptor)-negative patients, EGFR−- and CD44−-positive patients both experienced shorter DFS (*p* = 0.044, *p* = 0.063) and OS (*p* = 0.016, *p* = 0.038; Fig. 5). In HR-positive subtype, CD24-positive patients had significantly worse DFS (*p* < 0.05, Fig. 6). In EGFR-/CD44-stratified analysis, patients with EGFR+/CD44+ subtype had the worst prognosis, patients with EGFR/CD44 single positive subtype followed, patients with EGFR−/CD44− subtype had the best prognosis (Fig. 7).

## Discussion

IDC is the most common type of breast cancer. BIDC can be divided into triple-negative and non-triple-negative based on the hormone receptor status. TNBC accounts for 10–17 % of all breast cancers [15]. As we studied IDC in the present study, TNBC only accounted for 8.5 % of all breast cancers.

TNBC is relatively large, high grade, has a high rate of node positivity at diagnosis, and is biologically more aggressive. Despite the higher rates of clinical response to neoadjuvant chemotherapy, TNBC patients have a higher rate of distant recurrence and poorer prognosis compared with patients with other breast cancer subtypes [2, 3]. Consistent with the literature, our findings demonstrated that TNBC patients had significantly worse DFS and OS compared with non-TNBC patients (Fig. 2a, e).

EGFR is expressed in all types of breast cancer, especially in TNBC [5, 16]. EGFR was first reported as a significant prognostic factor in breast cancer by Sainsbury et al. [17]. Since then, many researchers have reported differing findings on the prognostic value of EGFR in breast cancer, and there is still no agreement on the relationship between EGFR and clinical outcome [18, 19]. In the present study, EGFR expression was inversely correlated with ER status (*p* = 0.023). Although EGFR was frequently expressed in TNBC, there was no statistically significant difference between TNBC and non-TNBC patients (*p* = 0.108). EGFR-positive patients were more likely to develop distant metastases (*p* = 0.018), which indicated that EGFR may be an important prognostic factor for distant metastasis. Survival analysis showed that EGFR positive indicated significantly shorter survival time in the lymph node- and HR-negative group. These data strongly indicate that EGFR-positive status is related to breast cancer progression and is a pivotal prognostic factor for specific subgroup.

Although EGFR-positive status is correlated with poor clinical prognosis, Tang et al. [20] found that EGFR overexpression predicted better response to neoadjuvant chemotherapy in patients with TNBC. This is a possible reason that TNBC is more chemosensitive.

Numerous studies have reported that CD44+/CD24− epithelial tumor cells are the most common in TNBC [14, 21, 22], but the relationship between CD44+/CD24− cells and prognosis in breast cancer is debated. Lee et al. reported that a high proportion of CD44+/CD24− tumor cells in prechemotherapy tissue was correlated with higher histological grade, ER negativity, and high Ki-67 proliferation index. After primary systemic therapy, the proportions of CD44+/CD24− tumor cells were significantly increased [23]. Wu et al. [14] demonstrated that the CD44+/CD24− and CD24+/CD44− phenotypes associated with decreased DFS but were not independent predictors for DFS. Giatromanolaki et al. [22] reported that the CD44+/CD24− and CD44−/CD24− phenotypes indicated unfavorable prognosis in TNBC. However, Kim and colleagues reported that the CD44+/CD24− group is considered a favorable prognostic subgroup in breast cancer. CD24 expression was a poor prognostic marker in HR-positive breast cancer, and CD44 expression was a good prognostic marker in the HR-negative group [24]. Another study showed that there was no correlation between the CD44+/CD24− phenotype and outcome in HR-positive breast cancer [25]. In the present study, 76.9 and 50 % of TNBC and non-TNBC patients, respectively, showed positive CD44 expression (*p* < 0.05). However, CD24 did not show statistically different expression levels between the two groups (*p* = 0.131). CD44-positive expression was inversely associated with HER2-positive status and HR-positive status, while CD24-positive expression was correlated with HER2-positive status but had nothing to do with HR-positive status. This study showed that CD44 positive was significantly correlated with survival time only in lymph node- and HR-negative patients while CD24 positive was significantly correlated with survival time in lymph node-negative and HR-positive patients.

Although the rate of EGFR expression is high in breast cancer, especially in TNBC, EGFR inhibitors do not have an ideal curative effect in breast cancer. Previous studies have suggested that synthetic lethal cross-linking of inhibitors of the mitogen-activated protein kinase (MAPK) kinase (MEK)/MAPK pathway, EGFR, and poly(ADP-ribosyl) transferase (PARP) may be related [26, 27]. In this study, EGFR and CD44 expressions were significantly positively correlated (*p* = 0.017). The CD44+/CD24− tumor cell phenotype is recognized as a cancer stem cell characteristic [11]. It is believed that CD44+/CD24− breast cancer cells are highly invasive and radioresistant and chemoresistant [28]. The close correlation between CD44 and EGFR expressions may be another reason for the resistance to EGFR inhibitors in breast cancer. Combining EGFR inhibitors with CD44 inhibitors may be a novel method for breast cancer treatment, especially TNBC.

In conclusion, our study indicates that in combination, EGFR and CD44/CD24 expression status are powerful identifiers of breast cancer patient subgroups with different clinical behavior. Although our study has some limitations, such as its retrospective design and relatively small number of studied patients, the data obtained indicate that EGFR and CD44 could serve as useful biomarkers for better determination of the prognosis of invasive breast cancer. These findings may have therapeutic significance and may improve the management of breast cancer patients.

## References

[1] Network Cancer Genome Atlas (2012). Comprehensive molecular portraits of human breast tumours. Nature.

[2] Dent R, Trudeau M, Pritchard KI, Hanna WM, Kahn HK, Sawka CA (2007). Triple-negative breast cancer: clinical features and patterns of recurrence. Clin Cancer Res.

[3] Haffty BG, Yang Q, Reiss M, Kearney T, Higgins SA, Weidhaas J (2006). Locoregional relapse and distant metastasis in conservatively managed triple negative early-stage breast cancer. J Clin Oncol.

[4] Chen JQ, Russo J (2009). ER alpha-negative and triple negative breast cancer: molecular features and potential therapeutic approaches. Biochim Biophys Acta.

[5] Rakha EA, El-Sayed ME, Green AR, Lee AH, Robertson JF, Ellis IO (2007). Prognostic markers in triple-negative breast cancer. Cancer.

[6] Guillamo JS, de Bouard S, Valable S, Marteau L, Leuraud P, Marie Y (2009). Molecular mechanisms underlying effects of epidermal growth factor receptor inhibition on invasion, proliferation, and angiogenesis in experimental glioma. Clin Cancer Res.

[7] Nielsen TO, Hsu FD, Jensen K, Cheang M, Karaca G, Hu Z (2004). Immunohistochemical and clinical characterization of the basal-like subtype of invasive breast carcinoma. Clin Cancer Res.

[8] Finn RS, Press MF, Dering J, Arbushites M, Koehler M, Oliva C (2009). Estrogen receptor, progesterone receptor, human epidermal growth factor receptor 2 (HER2), and epidermal growth factor receptor expression and benefit from lapatinib in a randomized trial of paclitaxel with lapatinib or placebo as first-line treatment in HER2-negative or unknown metastatic breast cancer. J Clin Oncol.

[9] Buchholz TA, Tu X, Ang KK, Esteva FJ, Kuerer HM, Pusztai L (2005). Epidermal growth factor receptor expression correlates with poor survival in patients who have breast carcinoma treated with doxorubicin-based neoadjuvant chemotherapy. Cancer.

[10] Tsutsui S, Ohno S, Murakami S, Hachitanda Y, Oda S (2002). Prognostic value of epidermal growth factor receptor (EGFR) and its relationship to the estrogen receptor status in 1029 patients with breast cancer. Breast Cancer Res Treat.

[11] Ponti D, Costa A, Zaffaroni N, Pratesi G, Petrangolini G, Coradini D (2005). Isolation and in vitro propagation of tumorigenic breast cancer cells with stem/progenitor cell properties. Cancer Res.

[12] Al-Hajj M, Wicha MS, Benito-Hernandez A, Morrison SJ, Clarke MF (2003). Prospective identification of tumorigenic breast cancer cells. Proc Natl Acad Sci USA.

[13] Honeth G, Bendahl PO, Ringner M, Saal LH, Gruvberger-Saal SK, Lovgren K (2008). The CD44+/CD24− phenotype is enriched in basal-like breast tumors. Breast Cancer Res.

[14] Wu Y, Sarkissyan M, Elshimali Y, Vadgama JV (2013). Triple negative breast tumors in African-American and Hispanic/Latina women are high in CD44+, low in CD24+, and have loss of PTEN. PLoS One.

[15] Reis-Filho JS, Tutt AN (2008). Triple negative tumours: a critical review. Histopathology.

[16] Burness ML, Grushko TA, Olopade OI (2010). Epidermal growth factor receptor in triple-negative and basal-like breast cancer: promising clinical target or only a marker?. Cancer J.

[17] Sainsbury JR, Farndon JR, Needham GK, Malcolm AJ, Harris AL (1987). Epidermal-growth-factor receptor status as predictor of early recurrence of and death from breast cancer. Lancet.

[18] Zhang M, Zhang X, Zhao S, Wang Y, Di W, Zhao G et al. Prognostic value of survivin and EGFR protein expression in triple-negative breast cancer (TNBC) patients. Target Oncol. 2013. doi:10.1007/s11523-013-0300-y. 10.1007/s11523-013-0300-y 24233638

[19] Klijn JG, Berns PM, Bontenbal M, Alexieva-Figusch J, Foekens JA (1992). Clinical breast cancer, new developments in selection and endocrine treatment of patients. J Steroid Biochem Mol Biol.

[20] Tang Y, Zhu L, Li Y, Ji J, Li J, Yuan F (2012). Overexpression of epithelial growth factor receptor (EGFR) predicts better response to neo-adjuvant chemotherapy in patients with triple-negative breast cancer. J Transl Med.

[21] Idowu MO, Kmieciak M, Dumur C, Burton RS, Grimes MM, Powers CN (2012). CD44(+)/CD24(-/low) cancer stem/progenitor cells are more abundant in triple-negative invasive breast carcinoma phenotype and are associated with poor outcome. Hum Pathol.

[22] Giatromanolaki A, Sivridis E, Fiska A, Koukourakis MI (2011). The CD44+/CD24− phenotype relates to ‘triple-negative’ state and unfavorable prognosis in breast cancer patients. Med Oncol.

[23] Lee HE, Kim JH, Kim YJ, Choi SY, Kim SW, Kang E (2011). An increase in cancer stem cell population after primary systemic therapy is a poor prognostic factor in breast cancer. Br J Cancer.

[24] Kim HJ, Kim MJ, Ahn SH, Son BH, Kim SB, Ahn JH (2011). Different prognostic significance of CD24 and CD44 expression in breast cancer according to hormone receptor status. Breast.

[25] Hashimoto K, Shimizu C, Tsuda H, Saji S, Osaki A, Shigekawa T (2012). Immunohistochemical detection of breast cancer stem cells in hormone receptor-positive breast cancer and their role in response to endocrine therapy and clinical outcome. Oncol-Basel.

[26] Normanno N, De Luca A, Maiello MR, Campiglio M, Napolitano M, Mancino M (2006). The MEK/MAPK pathway is involved in the resistance of breast cancer cells to the EGFR tyrosine kinase inhibitor gefitinib. J Cell Physiol.

[27] Nowsheen S, Cooper T, Stanley JA, Yang ES (2012). Synthetic lethal interactions between EGFR and PARP inhibition in human triple negative breast cancer cells. PLoS One.

[28] McClements L, Yakkundi A, Papaspyropoulos A, Harrison H, Ablett MP, Jithesh PV (2013). Targeting treatment-resistant breast cancer stem cells with FKBPL and its peptide derivative, AD-01, via the CD44 pathway. Clin Cancer Res.

